# Freud’s Model of the Mind Within a Predictive Processing Neuroscientific Paradigm

**DOI:** 10.3390/e28030318

**Published:** 2026-03-12

**Authors:** Erik Stänicke, Bendik Sparre Hovet, Line Indrevoll Stänicke

**Affiliations:** 1 Department of Psychology, University of Oslo, Pb 1094 Blindern, 0317 Oslo, Norway; 2 Lovisenberg Hospital, Nic Waals Institute, Pb 4970 Nydalen, 0440 Oslo, Norway

**Keywords:** neuroscientific paradigm, prediction, projection, psychoanalysis

## Abstract

The recent paradigm shift within cognitive neuroscience toward predictive processing appears to align with many psychoanalytic conceptualizations of the mind. In this article, we argue that several psychoanalytic concepts, such as projection, transference, wish-fulfillment, and perceptual identity, are particularly compatible with the current neuroscientific conception of the brain as a prediction machine. Specifically, we propose that the concept of projection as used in modern psychoanalysis to explore subjective experience and fantasies is closely analogous to the concept of prediction as it is used to explain the fundamental cognitive functions of the brain. We discuss the implications of this parallelism for understanding the role of homeostasis in psychoanalysis and cognitive neuroscience, and we also discuss the parallels between insight and surprise in these two fields of mental science. Limitations in drawing parallels between projection and prediction are also addressed. By integrating these two fields, we envision the possibility of tackling subjectivity scientifically.

## 1. Introduction

In this article, we explore the proposition that Sigmund Freud’s model of the mind aligns with modern computational neuroscience. Psychoanalysis is often understood as synonymous with Freud’s theories, even though it has developed significantly beyond his original ideas. Nonetheless, some of Freud’s most basic ideas are still of central importance and are supported by empirical evidence from neuropsychology. As Carhart-Harris & Friston [1] point out, “the psychoanalytic distinction between the primary and secondary processes (as functions of the id and ego respectively) fit comfortably with modern notions of functional brain architecture, at both a computational and neurophysiological level” (p. 1265). In this article, we argue that Freud’s model of the mind which entails the mechanisms of perceptual identity, projection, transference, and wish fulfillment complements the paradigm of “predictive processing” within computational neuroscience. Furthermore, the use of projection and introjection in modern psychoanalysis serves the same purpose: to understand the human mind as a system that seeks homeostasis.

Freud [2] stated that the mind continuously works at the interface between inner and outer reality. Essential in the process of communication and alignment of psychical reality (with all its feelings, thoughts, and phantasies) on the one hand and material reality on the other is a process of the mind seeking to align current perceptions with past experiences that have fulfilled a wish, i.e., have been gratifying. Thus, the mind aims for perceptual identity [3] Seeking perceptual identity is another way of understanding the mind as conservative, i.e., that it aims to return to preferred states (this is homeostasis). As Freud [4] wrote in his paper “On Negation”, the infant first considers if what he experiences in the world gives pleasure or not. Only when he has evaluated whether something, such as the breast, gives pleasure or unpleasure, does he ask himself if he should continue seeking or avoiding it, and by doing so, asks himself if the breast is real. For Freud, this is a model of how the pleasure principle precedes the reality principle, which is more aptly explained in the concept of perceptual identity. The central point is that psychic equilibrium, homeostasis, comes before the reality principle. In what follows, we discuss whether the paradigm of predictive processing within modern computational neuroscience can explain what Freud and later psychoanalysts, such as Melanie Klein and Wilfred Bion, described from the vantage-point of subjective experience. We will argue that the psychoanalytic model and the predictive processing paradigm, respectively, describe and explain the mind as aiming for homeostasis on two levels of abstraction which nonetheless complement each other.

There are already others who have argued that the predictive processing paradigm shows promise in explaining psychotherapy generally [5,6,7,8,9,10,11,12,13,14,15,16,17,18,19] and psychoanalysis specifically [20,21,22,23,24,25,26]. However, none of these earlier works has, to our knowledge, provided an explanatory account of the connection, beyond pointing out the parallel. We will try to demonstrate more closely how the psychoanalytic model of the mind and the predictive processing paradigm complement each other.

## 2. The Psychoanalytic Model of the Mind: Projection and Introjection

From the beginning of Freud’s development of a psychoanalytic model of the mind, there was a premise that the mind seeks homeostasis—although Freud did not use the term (which was introduced by [27]). As mentioned in the Introduction Section, Freud’s [2] theory of perceptual identity portrays the mind as using past experiences as a prototype for fulfilling present needs. If the infant has been satisfied in one manner in the past, it will subjectively generate the same perception in the present. The hungry infant will unconsciously remember that the breast satisfied its hunger and hallucinate or seek out the presence of the breast. Perceptual identity does not limit itself to repressed desires but to all desires, making it the fundamental mechanism of wish fulfillment. As such, the goal of the pleasure principle is to generate perceptions that are identical to our desires. However, the reality principle, representing the demands of the external world, will of course constrain this possibility. Prior to the establishment of this principle, however, according to this early model of Freud, our primal mode of mental functioning is to hallucinate the objects that satisfy our needs [28]. This is a primary process that follows us throughout our lives, lurking underneath more mature psychological functioning. Freud never fully developed his theory of perceptual identity [3] but instead developed several other concepts such as wish-fulfillment, transference, and projection. With the concept of “wish-fulfillment,” Freud [2] claimed that humans can satisfy unacknowledged desires through fantasy and dreams. “Transference” is a concept that Freud [29,30,31] proposed for the clinical observation that the patient often had reactions toward the analyst based on relational expectations derived from prior experience.

Thus, all these concepts of mental functioning—perceptual identity, wish-fulfillment, and transference—portray a system that aims for homeostasis: a system that attempts to return to its preferred states. However, we will especially highlight the concepts of projection and introjection in this context because they have been further developed in modern psychoanalysis.

Projection and introjection as psychoanalytic concepts have a long history. Accordingly, how they have been understood has evolved during Freud’s long career, but also in the work of analysts after him, especially Klein and Bion. Let us begin with how the concepts were understood by Freud (see also [32]). He began using the term “projection” with reference to paranoia, where he claimed that these patients project their own aggressive intentions into the external world, with the effect that they become afraid of being attacked [33]. In this sense, projection is understood as a defense mechanism. In later texts, Freud [31] used a similar conceptualization to explain phobic symptoms, which he understood as a defense whereby an internal threat is “warded off” and experienced as a threat coming from the outer world, e.g., phobia for dogs. Another example is the defense against one’s own impulse to be unfaithful, projected as jealousy of one’s partner [34].

Sandor Ferenczi [35], who was of course a close colleague of Freud, argued that introjection is the counterpart of projection. Freud [31,34] made use of this distinction by claiming that we introject everything that is good into ourselves and project everything that is bad. This brings another layer to the concepts because they can now be understood as being the mechanisms that are on the frontier between an internal and external reality, or in other words, between the subjective and objective worlds. In this connection, Freud compared the mind to the esophagus, saying that our attention is naturally directed to the external world, and any attempts at changing this ‘direction of flow’ is strongly resisted [36]. These claims allude to a metapsychology that Freud never explicitly spelled out [32] but that inspired further developments in later psychoanalysis. For example, there have been discussions of what it is that is projected, i.e., is it feelings, thoughts or objects that are projected? Clinically, this will be the difference between understanding the patient as projecting anger onto others or the patient projecting a complex fantasy of an intersubjective interaction of the other being angry when they have a specific need of comfort.

Later theorists in psychoanalysis have further developed the theory of projection and introjection consisting in fantasies of object relations. This opens more complex projections and introjections, like the patient who has nourished fantasies of being bad in order to keep their mother as all good. This patient has projected all the good parts of himself into his mother and sacrificed himself by ending up with all the badness within himself.

An example of introjection may be a woman who, as a child, was frequently criticized by her strict mother. As an adult, she still hears an inner critical voice that says she is not good enough. When she gets praise, she quickly turns it down as undeserved. The example illustrates how the woman has internalized her mother’s vigilant and critical attitude; it has become part of her internal object world. This introjection stabilizes her inner world by making external threats of critique predictable.

Another meta-psychological ambiguity within Freud’s theory of these mechanisms is the question of projection and introjection presupposing the differentiation of internal and external reality. As Laplanche and Pontalis [32] argue, Anna Freud can be understood as thinking that projections are something that the infant can generate only after it has developed a rudimentary differentiation of the internal and external worlds: me and not me. Klein can be read as thinking that the mechanisms of projection and introjection constitute this distinction.

Klein [37] proposed a theory of the development of the mind consisting of projection and introjection as the main mechanisms. She stated that inner object relations are “molded” by these mechanisms. More precisely, she developed a specification of projective processes by seeing them as consisting not only in evacuation of the bad but also that the bad became identified within the other, hence, a process of “projective identification.” Still, this projective identification was, in Klein’s understanding, an intrapsychic mechanism, something that expressed itself in phantasies. With Bion [38] projective identification became understood as an intersubjective process, as a form of proto-communication. The infant communicates its states, say frustration, by crying and thereby arouses the significant other and motivates them to understand what is needed and to act accordingly. Later analysts following this tradition, such as Betty Joseph [39], specified that these mechanisms imply that the mind aims at attaining “psychic equilibrium”. Phantasies of evacuating bad objects and introjecting the good objects are thereby seen as stabilizers of the mind.

Hence, in the object relational theory, projection and introjection imply two essential claims: that we humans strive to foresee relations with others based on past experiences and that we seek equilibrium by trying to evade frustration and pain in relationships with others. Thus, the mechanisms of projection and introjection aim at predicting interactions with others which avoid unpleasant affects. The theory of these mechanisms, especially with projective identification, postulates that we humans even try to “push” others to comply with our expectations. From a psychoanalytic perspective, humans have a strong propensity to relate in the ways that we are familiar with. So, if someone invites us into a new form of interaction, we may get anxious. We can even try to get the other to fulfill our expectations by pressing them into roles that fit with our past experiences. Inner object relations function as templates of what to expect from the world, as we try to realize our anticipations to balance out uncertainty.

Additionally, projection and introjection influence all parts of our mind: the cognitive, affective, and behavioral. Projection in the cognitive register can be seen when we attribute opinions to others, such as seen in prejudices against out-groups of people, i.e., “it is them that are critical of us, so we have to defend ourselves.” Affective projections are already mentioned in Freud’s example of phobic anxiety: “I am afraid of dogs because they are aggressive.” Projection can also lead to actions, as specified by Bion’s theory of projective identification as a proto-communication. It claims that we behave in ways that stimulate reactions in others that can activate states, and thus behaviors in the other that meet our needs or even identify the bad in the other. In everyday psychology, we may have experience of becoming angry after our partner insists that we are angry. The main point here is that some of our expectations of others’ reactions towards us are not on a cognitive level and thus are difficult to identify and work with in therapy. In psychoanalysis, these mechanisms can be expressed in ways of being with the other and, thus, it can be worked with in the transference.

## 3. The Predictive Processing Paradigm

The predictive processing model is a paradigm of the mind where the brain’s main task is to construct and interact with the world through predictions [40]. It marks an expansion from a traditional stimulus (S) → organism (O) → response (R) model of the mind represented in, for example, information processing theory [41]. In this earlier model, perception is generated by bottom–up stimuli. However, it has become increasingly clear that mental representations and internal models of the world influence how we perceive and act in the world [42]. The main task of the brain, as presented in the modern paradigm, is to match incoming sensory stimuli with probabilistic top–down generative expectations based on prior experience and inherent biological imperatives [40].

We start with innate predictions about biologically significant situations. A common example for humans would be the fear of snakes [43]. We expect that snakes are dangerous, and the accompanying feeling when seeing one is fear. Then, we constantly update our outgoing predictions based on experience gained from former incoming prediction errors. If you are exposed to non-dangerous snakes, you might stop being afraid of snakes after the new experience has proven the inherent prediction wrong. To prove that the snake is not dangerous, you might have to pick it up or at least step close to it, which means predictions are about actions as much as they are about perception. But if the prediction that the snake is dangerous is strong enough, you will never pick it up in the first place, thus upholding your prediction of the snake as dangerous. This is called “active inference” where perception and action are two sides of the same predictive coin. To perceive is to predict, to act is to act out the prediction. We can test our predictions, and potentially update them, or act to ensure they remain true, such as avoiding situations that might contradict them. There is constant feedback between prediction signals from higher levels of the internal model of the world and lower levels of experience from the incoming sense data, making the model hierarchical [44,45]. Error signals are triggered when the top–down and bottom–up information is in conflict: when our predictions do not work. This hierarchical predictive processing functions ideally by updating our predictive models based on prediction error, thus creating a more accurate model to navigate the world [46]. The world we perceive and interact with is a world that we create from our past, from expectancies and actions.

Even earlier versions of the information processing theory contained elements of top-down prediction, most notably what was called the “analysis-by-synthesis” tradition in cognitive psychology. This was in part based on Helmholtz’s [47] insight that perception is based on how we expect the world to be, i.e., predictions. For Helmholtz, our sensory systems must infer the cause of incoming signals to bridge the gap between our internal and external worlds. To accomplish this difficult task, the cause of our external world is inferred by probabilistic models. As revealed in his description of this process as “unconscious interference”, Helmholtz [48] already thought of predictions as happening below conscious awareness. Prediction as a fundamental way to perceive the world was expanded upon by Mackay [48], Neisser [49], and Gregory [50]. However, these elements of “analysis-by-synthesis” have largely been neglected within the information processing paradigm, and an overview of the information processing approach only mentions Neisser in passing as an example of the above authors [51]. Still, the idea of predictions as being fundamental for the mind is not new.

What is the basis for proposing that the essential function of the mind is minimizing prediction error? One way of anchoring predictions anatomically and physiologically is to conceptualize them as the brain’s way to minimize the ‘free energy’ of information, known as “the free energy principle” [52,53]). All biological systems seek to reduce entropy, the constant tendency of matter to dissolve into disorder. This is no different for the human mind [54]. Entropy for the predictive brain is the average free energy given the information it has about the world. Bottom–up input, such as seeing a tree, is bound by top–down models of the world, called priors, such as the knowledge that the tree is common in the forest you are walking through [55].

To bind bottom–up input through priors is to minimize free energy, which is felt as minimizing surprise. This is the brain doing useful work. To reduce free energy, the mind constantly tries to work out the difference between how the world is and how we expect it to be. We have two fundamental ways of reducing free energy: perceptual inference and active inference [56]. With perceptual inference, we update our predictive model to better match the sensory input, and with active inference, we act on the world to make the sensory input better match our predictive model. When the world meets our expectations, or we manage to make it meet our expectations, we minimize free energy, which moves us away from entropy and closer to homeostasis [28]. Hence, making accurate prediction models answers a fundamental need to decrease entropy [57].

When the world does not match our expectations, we feel surprised [58]. Surprise is an affective signal of our model of the world becoming more disordered, increasing entropy and free energy—more technically termed “surprisal” [59]. As such, surprisal is a signal of uncertainty and is felt as unpleasure [58,60]. From the clinic, we can think it is peculiar if a patient with borderline functioning resists a trusting relationship with a good therapist. However, understood in this context, we can say that the patient feels surprised, and thus unpleasure, with meeting a person, the therapist, that does not conform to their expectation. Pleasure and pain feel like something to force us to either update our internal models or act in the world to make it conform to our expectations [58,61]. Thus, unpleasurable and pleasurable feelings arise with increasing or decreasing prediction errors. This can also be seen in the dopamine reward from prediction error coding when we get more reward than expected, more dopamine, or less reward than predicted, less dopamine [62]. Pleasure and unpleasure give us information about when the world adheres to our expectations and when it does not. In the predictive processing paradigm, feelings let us know when we should update our predictions. Updating our predictions about the world in line with our feelings is thus the fundamental *work* of the mind.

Several authors have already pointed out how the free energy principle aligns with and can explain elements of psychoanalysis [21,22,63,64]. As mentioned above, Freud understood the mind and the nervous system through the concept of homeostasis, decades before the term was coined [64]. Freud [31] understood this principle to be measured in the central nervous system, as indicated by him writing the nervous system is an apparatus having the function of abolishing stimuli. There is circumstantial evidence for the free energy principle, for example, that there are more neural fibers reaching the eye from the brain than there are neural fibers extending from the eye to the visual cortex (20). Further, it has been pointed out that interoceptive inference can be translated neuroanatomically into, e.g., the way the visceromotor areas (VMAs) issue predictions that serve as homeostatic set points, which further ascends into the subcortex, brainstem, and spinal cord, to areas such as the periaqueductal gray (PAG) [65]. Further, the two fundamental ways of reducing free energy by perceptual inference and active inference closely mirror the psychoanalytic concepts of autoplastic and alloplastic adaptation, which we will expand on in the Discussion Section. However, this paper does not aim to ground psychoanalytic projection and introjection completely in the mathematical formalism of the free energy principle. Rather, it points to where predictive coding and psychoanalytic concepts, such as perceptual identity, wish-fulfilments, transference, and introjection and projection, share a common conceptual architecture aimed at homeostasis. Psychoanalysis offers a descriptive phenomenology of what predictions feel like from the inside, which mathematical and neuroscientific disciplines do not concern themselves with directly.

## 4. Discussion

We have presented the basic psychoanalytic theory of the mind and argued that it plays a central role in the mechanism for upholding homeostasis. Historically, the understanding of psychological processes, especially projection and introjection, has been clinically refined after Freud. The main goal is for the human mind to feel prepared by having fantasies of what to expect from others, based on past experiences of relationships. Furthermore, the mechanisms strive toward homeostasis, i.e., that we seek out the familiar and, thus, regulate anxiety and feel safety. By projecting the bad onto others and introjecting the good into us, we may feel threatened, but at least it is a threat that is recognizable and expected. Then, we presented the predictive processing paradigm within cognitive neuroscience. This model claims that the human brain works by making predictions about significant situations. Thus, we make probabilistic models of ourselves in the world based on experience and inherent biological imperatives. These models are constantly updated to fit with the world. When our predictions fail, they activate feelings. Thus, feelings are a signal that we cannot meet the psychological or physical challenges as expected, and thus demand further mental work to find better predictions which can provide better solutions in the world.

Within predictive processing, a lack of alignment between predictions and external reality, which spurs surprisal, can be resolved in two ways. First, through perceptual inference, the person updates their internal model to fit external reality better. In a psychoanalytic treatment this would consist of nuancing inner objects that provide more mature self-care. Second, by active inference, the person acts on the world so that it conforms to its existing model. Within a psychoanalytic perspective, this will consist of the person making use of defensive strategies of denial, projective identification, or other ways to push reality to conform to one’s inner model. These two routes to error minimization are psychoanalytically explained by introjection and projection, respectively, and consist of what we mentioned as autoplastic and alloplastic strategies.

Our two clinical examples of paranoid projection and the woman with an internalized critical voice of her mother can be understood as rigid uses of the second route. An experience of others as hostile or having a self-critical inner voice is preserved, while external misalignment with this is construed as still persecutory or undeserved. The one with paranoia and the one with a self-critical inner voice minimize surprise at the cost of reality testing and psychological flexibility. It must be underlined that some patients, such as those with predominantly avoidant relational strategies, may need help to not only revise their inner models but also to test in action whether external objects can respond more benignly than expected. Explorative forms of active inference in relations can also enable more flexible updating of internal object relations.

The predictive paradigm holds great promise within neuropsychological research. It portrays the human being as proactive in engaging with the world, making predictions of what will happen based on past experiences. This is quite different from the old information processing paradigm, which portrayed the mind as reactive to stimuli. It has been argued convincingly that the predictive paradigm implies a vision of the mind that has affinities to the philosophy of Immanuel Kant, with its top–down generation of precepts [66]. In our opinion, the model can also be understood as being aligned with philosophical conceptualizations such as Martin Heidegger’s [67] theory of the human being as *always already* invested and engaged with its world, so much so that he labeled our existence as a “being-in-the-world.” However, unlike Kant’s transcendental epistemology and Heidegger’s existential ontology, the predictive paradigm of the brain explains *how* the mind works to be actively engaged with the world. And it is the question of how the mind works that makes psychoanalysis especially relevant, but, as we will argue, its contribution is on a phenomenological level. Even if psychoanalysis is a clinical enterprise that is developed in the therapy room with the patient on the couch as its base for data, it still has a closer affinity to modern brain research than ever before in accordance with Freud’s early aim [29].

As presented, the concepts of wish-fulfillment, transference, and projection are all defense mechanisms in that their main function is to protect the mind from dealing with unpleasant feelings. Transference and projection protect by placing or evacuating feelings outside us onto or into persons, and wish-fulfillment by having the need fulfilled within oneself. Maybe a more precise parallel to predictions within the neuroscientific paradigm is Freud’s [2] early concept of perceptual identity, as it pertains directly to how we create perceptions of the world based on our prior experiences of satisfaction. It explains how the mind aims at reaching homeostasis by itself. But as Freud argued, the reality principle, the demands of the external world, distorts this and makes a demand on the ego to do work to find satisfaction. Furthermore, the concept of perceptual identity was never fully developed [3] Instead, modern psychoanalysts further developed the concepts of projection and introjection, and we will state now that they were implicitly informed by the idea of perceptual identity. Thus, projection can be understood as psychoanalysis’s counterpart to neuroscience’s concept of prediction. Projections are the mind’s way of trying to uphold homeostasis by evacuating unpleasant feelings, but when it does not work—when reality resists or does not comply—then the mind must do work and try to expand its internal model, in psychoanalysis called internal “object relations”, by introjecting new experiences.

Both perceptual identity and the free energy principle aim towards homeostasis by creating a similarity between past and present. In perceptual identity, the world is perceived according to how it has previously met a need. An everyday example, apart from the defense mechanisms already mentioned, is the unconscious tendency towards repetition compulsion, that we repeat our negative patterns, because they are familiar and thus acting them out keeps us in homeostasis [68]. More fundamentally, when the pleasure and reality principle do not match, we can either react by trying to change our internal reality, which is autoplastic adaption, or the external reality, alloplastic adaption [35,69]. Too much free energy, felt as unpleasure, is reduced through similar conservative mechanisms. With perceptual inference, the perception of the world, the prediction, is updated, similar to autoplastic adaption. With active inference, a rigorous and unflexible prior can make one deny or change aspects of reality to accommodate the prediction. Active inference mirrors alloplastic adaption, and may take the form of projective identification, denial, or other defense mechanisms. Together, perceptual identity and the free energy principle explain how maladaptive patterns can emerge from two levels of analysis. One can stay in an abusive relationship, while ignoring the evidence that it is abusive, to maintain perceptual identity by denying the abuse as an alloplastic adaption. On a neurological level, it takes work to bind increased free energy if one has to update the prediction of being safe, so the brain ignores data that challenge this prior.

We think it is proper to understand these different models of the mind as studying the same phenomenon but from different perspectives. Cognitive science studies the basic mechanisms in the brain that have evolved for us as a species to survive. Thus, it aspires to seek objective knowledge that can explain human behavior on a general level. Psychoanalysis, on the other hand, has for over a century studied the human mind from a subjective point of view. We contend that psychoanalysis studies, without stating it explicitly, the human mind as a prediction machine, but it is studied introspectively, as it is experienced. Thus, psychoanalysis provides the content of predications, how a predication is felt.

Specifically, modern psychoanalysis works with the patient’s projections as they often are expressed in the transference towards the analyst, which may be explained as predictions. Thus, psychoanalysis is especially attuned to the study of the predictive mind in relational situations. Projection is a concept that refers to the content of predictions: if my brain makes a prediction that I have to show off in social situations in order to fight my way up the social hierarchy, then my phenomenological projections may consist of fantasies of my rival being a person who does not deserve its place in the social group and that I will do the group a favor if I humiliate him. Hence, we contend that the neuropsychological theory of predictive processing explains why we humans project our feelings, cognitions, or actions onto others—because projections aim at decreasing entropy and maintaining equilibrium. As we wrote above, Joseph [39] claimed that the mind, according to psychoanalysis, seeks equilibrium. However, she lacked a neuroscientific reason for stating this. She based her claim solely on the observation that we can actually see it, i.e., that patients are willing to uphold negative relationships if they confirm expectations based on past experience. Even Freud was in his later theories concerned with the feeling of homeostasis, which Freud called the Nirvana principle [28,70,71]. But while Freud thought the Nirvana principle was “beyond” the pleasure principle, the goal of pleasure is to lead us toward a state of no need [63]. To actually reach the state of no need, our predictions about the world must be without error.

Another noteworthy conjunction between the two paradigms of the mind is the role that surprise and insight play in the two. As we presented earlier, surprise is an effect of prediction error, and thus highly significant, since it activates feelings that motivate the individual to recognize their failure and make better predictions [58]. In psychoanalysis, insight has been understood as a central mechanism of change. We can, with this prediction theory, now understand it in a more nuanced fashion and understand why insight plays such a central role. In a psychoanalytic treatment, one works with the patient’s transference to their analyst. Insight is, in this context, understood as instigating change when the patient sees how their relational patterns repeat themselves toward the analyst. With our new theories, we can understand this as the patient coming to grips with relational predictions towards the analyst which do not match what is happening, which leads to surprise and feelings.

It must be mentioned that there are, of course, several differences between the prediction paradigm and psychoanalysis, and one significant one will be highlighted here. The former has concerned itself with studying prediction errors, which leads to surprise and feelings that motivate making amendments to the predictions. An overall interpretation is that the mind makes predictions to increase the survival probability of the species. On the other hand, psychoanalysis has focused mainly on projections that we typically observe in psychopathology. While the former usually uses examples from everyday life, psychoanalysis uses illustrations from pathological states such as psychosis, personality disorders, or mood disorders. Thus, in psychoanalysis, projections can be understood as the individual patient’s attempt to make predictions that increase the probability of psychological survival and safety given an adverse social environment. It must also be underlined that psychoanalysis is a normative theory and practice that aims to explain mental illness and help amend it, while the prediction paradigm is mainly descriptive with the aim to explain the mind, not to heal.

We think that psychoanalysis has normative implications, for example, of what is a good outcome of treatment, what are good inner objects and safe attachments, and that the prediction model can mainly explain this formally and physiologically. However, we think that the prediction model highlights the need for therapy to work with predictions, not only cognitively, but also how it expresses itself in relationships, and that processes of change must imply surprises.

Yet it should be stressed that modern psychoanalysis is no longer a theory of only the darkest reaches of the mind. With object relations theory, and what has been termed the “relational turn”, psychoanalysis has come to put a greater focus on non-pathological psychic phenomena, such as love, trust, and play [72].

A further possible point of contention is that for predictive processing, all mental states are in essence predictions [73]. A wish is only a result of a web of prior predictions. Predictive processing thus dissolves the distinction between beliefs and desires [74]. It has been pointed out that this may run counter to more basic views of emotions, such as that of “Pankseppian” affective neuroscience [75]. This is where Friston’s Free Energy principle makes the innate subcortical emotions proposed by Panksepp possible to integrate with a predictive model of the cortex [63]. The fundamental goal of the mind, and of predictions, is still homeostasis [2,60]. And predictions are not random, but intrinsically motivated by our desires and ensuing wishes, motivating the startling phrase: “all thinking is wishful-thinking” [76].

While for psychoanalysis the bridges between the inner world and the outer world are projections and introjection, for predictive processing this has been speculated to be introjection in the form of interoceptive inference [77]. In line with what Friston termed “a duet for one”, however, our predictions must rely on relations to other human beings: intersubjectivity [78]. To specify the phenomenology behind how our predictions are in essence relational is thus an insight that psychoanalysis contributes to predictive processing [21].

Thus, we contend that psychoanalysis provides descriptions of how predictions work on our subjective experience. In psychoanalytic treatment, the analyst explores together with the patient their images, fantasies, affects, and relational expectations, and how these experiences “feel” and express themselves in the therapeutic relationship. On the other hand, predictive processing and its theories of free energy and active inference provide formal and physiological explanations of how the brain reduces surprise by making, testing, and updating hierarchical predictions.

## 5. Conclusions

We have argued that the predictive processing paradigm provides a mechanistic explanation, and the modern psychoanalytical model of projection provides a phenomenological understanding of the same phenomenon. While psychoanalysis could be argued to not have been on a par with its time while the information processing paradigm in neuropsychology was dominant, we think the prediction paradigm opens a new possible exchange and collaboration between the two disciplines. Psychoanalysis consists of a vast resource of theories about the subjective mind, projection being central among these, and neuro-psychoanalysis adds an external view of the mind while studying the biological and neurological substrate of psychology. Today, we see the possibility that the combination can potentially “tackling subjectivity scientifically” [79]. Psychoanalysis provides the normative aims of what to change, whereas predictive processing explains the mechanisms of how change occurs in the brain. This implies that therapy should target maladaptive predictions, adjusting the balance from rigid, defensive forms of active inference towards more flexible perceptual inference. Relational encounters in a safe environment, as in psychoanalysis, can produce surprise, which again permits new ways of predicting and being in the world.

## Data Availability

Data is contained within the article.

## References

[1] Carhart-Harris R.L., Friston K.J. (2010). The default-mode, ego-functions and free-energy: A neurobiological account of Freudian ideas. Brain J. Neurol..

[2] Freud S. (1900). Interpretations of Dreams.

[3] Sandler J. (1976). Dreams, unconscious fantasies and identify of perception. Int. Rev. Psychoanal..

[4] Freud S. (1925). Negation. Int. Rev. Psycho-Anal..

[5] Bissell D.A., Ziadni M.S., Sturgeon J.A. (2018). Perceived injustice in chronic pain: An examination through the lens of predictive processing. Pain Manag..

[6] Chamberlin D.E. (2019). The Predictive Processing Model of EMDR. Front. Psychol..

[7] Griffin J.D., Fletcher P.C. (2017). Predictive Processing, Source Monitoring, and Psychosis. Annu. Rev. Clin. Psychol..

[8] Fabry R.E. (2020). Into the dark room: A predictive processing account of major depressive disorder. Phenomenol. Cogn. Sci..

[9] Gadsby S., Hohwy J., Mendonca D., Curado M., Gouveia S. (2021). Why use predictive processing to explain psychopathology? The case of anorexia nervosa. The Philosophy and Science of Predictive Processing.

[10] Herzog P., Kube T., Fassbinder E. (2022). How childhood maltreatment alters perception and cognition—The predictive processing account of borderline personality disorder. Psychol. Med..

[11] Krupnik V. (2020). Trauma or Drama: A Predictive Processing Perspective on the Continuum of Stress. Front. Psychol..

[12] Kube T., Berg M., Kleim B., Herzog P. (2020). Rethinking post-traumatic stress disorder—A predictive processing perspective. Neurosci. Biobehav. Rev..

[13] Lutz A., Mattout J., Pagnoni G. (2019). The epistemic and pragmatic value of non-action: A predictive coding perspective on meditation. Curr. Opin. Psychol..

[14] Papalini S., Beckers T., Vervliet B. (2020). Dopamine: From prediction error to psychotherapy. Transl. Psychiatry.

[15] Putica A., Felmingham K., Garrido M.I., O’Donnell M.L., van Dam N.T. (2020). A predictive coding account of value-based learning in PTSD: Implications for precision treatments. Neurosci. Biobehav. Rev..

[16] Smith R., Badcock P., Friston K. (2021). Recent advances in the application of predictive coding and active inference models within clinical neuroscience. Psychiatry Clin. Neurosci..

[17] Van de Cruys S., Van Dessel P. (2021). Mental distress through the prism of predictive processing theory. Curr. Opin. Psychol..

[18] Wilkinson S. (2014). Accounting for the phenomenology and varieties of auditory verbal hallucination within a predictive processing framework. Conscious. Cogn. Int. J..

[19] Wilkinson S., Dodgson G., Meares K. (2017). Predictive Processing and the Varieties of Psychological Trauma. Front. Psychol..

[20] Holmes J. (2022). Friston’s free energy principle: New life for psychoanalysis?. BJ Psychol. Bull..

[21] Holmes J. (2024). Friston, Free Energy, and Psychoanalytic Psychotherapy. Entropy.

[22] Holmes J., Nolte T. (2019). “Surprise” and the Bayesian Brain: Implications for Psychotherapy Theory and Practice. Front. Psychol..

[23] Koslowski M., de Haas M.P., Fischmann T. (2023). Converging theories on dreaming: Between Freud, predictive processing, and psychedelic research. Front. Hum. Neurosci..

[24] McVey L., Nolan G., Lees J. (2020). The predictive moment: Reverie, connection and predictive processing. Br. J. Guid. Couns..

[25] Sands S. Predictive Processing: An Illuminating New Framework for Psychotherapy. Psychology Today, 30 June 2022. https://www.psychologytoday.com/us/blog/well-being-through-lifetime/202206/predictive-processing-illuminating-new-framework.

[26] Solms M. (2026). The Only Cure: Freud and the Neuroscience of Mental Healing.

[27] Cannon W. (1932). The Wisdom of the Body.

[28] Solms M. (2013). The Conscious Id. Neuropsychoanalysis.

[29] Freud S. (1895). Project for a Scientific Psychology.

[30] Freud S. (1912). The Dynamics of Transference.

[31] Freud S. (1915). Instincts and Their Vicissitudes.

[32] Laplanche J., Pontalis J.B. (1973). The Language of Psycho-Analysis.

[33] Freud S. (1896). Draft K. The Neurosis of Defence.

[34] Freud S. (1922). Some Neurotic Mechanisms in Jealousy, Paranoia and Homosexuality.

[35] Ferenczi S., Suttie J.I. (1919). The phenomena of hysterical materialization. Further Contributions to the Theory and Technique of Psycho-Analysis.

[36] Freud S. (1929). Letter to Einstein.

[37] Klein M. (1975). The Psychoanalysis of Children.

[38] Bion W.R. (1961). Experiences in Groups and Other Papers.

[39] Joseph B., Feldman M., Spillius E.B. (1989). Psychic equilibrium and psychic change. Selected Papers of Betty Joseph.

[40] Clark A. (2016). Surfing Uncertainty.

[41] Hutchinson J.B., Barrett L.F. (2019). The Power of Predictions: An Emerging Paradigm for Psychological Research. Curr. Dir. Psychol. Sci..

[42] Clark A. (2013). Whatever next? Predictive brains, situated agents, and the future of cognitive science. Behav. Brain Sci..

[43] Panksepp J. (1998). Affective Neuroscience: The Foundations of Human and Animal Emotions.

[44] Adams R.A., Shipp S., Friston K.J. (2013). Predictions, not commands: Active inference in the motor system. Brain Struct. Funct..

[45] Barrett L.F., Simmons W.K. (2015). Interoceptive predictions in the brain. Nature reviews. Neuroscience.

[46] Friston K.J., FitzGerald T., Rigoli F., Schwartenbeck P., Pezzulo G. (2017). Active Inference: A Process Theory. Neural Comput..

[47] Helmholtz H.V. (1860). Handbuch der physiologischen Optik. Handbook of Physiological Optics.

[48] Mackay D.M. (1956). Towards an information-flow model of human behavior. Br. J. Psychol..

[49] Neisser U. (1967). Cognitive Psychology.

[50] Gregory R.L. (1980). Perceptions as hypotheses. Philosophical transactions of the Royal Society of London. Ser. B Biol. Sci..

[51] Proctor R.W., Vu K.-P.L. (2006). The Cognitive Revolution at Age 50: Has the Promise of the Human Information-Processing Approach Been Fulfilled?. Int. J. Hum.–Comput. Interact..

[52] Friston K., Kiebel S. (2009). Predictive coding under the free-energy principle. Philosophical transactions of the Royal Society of London. Ser. B Biol. Sci..

[53] Friston K. (2010). The free-energy principle: A unified brain theory?. Nat. Rev. Neurosci..

[54] Friston K. (2009). The free-energy principle: A rough guide to the brain?. Trends Cogn. Sci..

[55] Kirchhoff M., Parr T., Palacios E., Friston K., Kiverstein J. (2018). The Markov blankets of life: Autonomy, active inference and the free energy principle. J. R. Soc. Interface.

[56] Parr T., Markovic D., Kiebel S.J., Friston K.J. (2019). Neuronal message passing using Mean-field, Bethe, and Marginal approximations. Sci. Rep..

[57] Feldman H., Friston K.J. (2010). Attention, uncertainty, and free-energy. Front. Hum. Neurosci..

[58] Solms M. (2024). Überraschung in Neurowissenschaft und Psychoanalyse. Jahrb. Psychoanal..

[59] Tribus M. (1961). Thermostatics and Thermodynamics: An Introduction to Energy, Information and States of Matter, with Engineering Applications.

[60] Solms M. (2019). The Hard Problem of Consciousness and the Free Energy Principle. Front. Psychol..

[61] Solms M., Friston K. (2018). How and why consciousness arises: Some considerations from physics and physiology. J. Conscious. Stud..

[62] Schultz W. (2016). Dopamine reward prediction error coding. Dialogues Clin. Neurosci..

[63] Solms M. (2018). Review of Damasio, the strange order of things. J. Am. Psychoanal. Assoc..

[64] Walker N. (1956). Freud and Homeostasis. Br. J. Philos. Sci..

[65] Seth A.K., Friston K.J. (2016). Active interoceptive inference and the emotional brain. Philos. Trans. R. Soc. Lond. B Biol. Sci..

[66] Swanson L.R. (2016). The Predictive Processing Paradigm Has Roots in Kant. Front. Syst. Neurosci..

[67] Heidegger M. (1927). Sein und Zeit [Being and time]. Jahrb. Für Philos. Und Phänomenologische Forsch..

[68] Freud S. (1914). Remembering, Repeating, and Working-Through.

[69] Freud S. (1924). The Loss of Reality in Neurosis and Psychosis.

[70] Freud S. (1920). Beyond the Pleasure Principle.

[71] Solms M. (2018). The neurobiological underpinnings of psychoanalytic theory and therapy. Front. Behav. Neurosci..

[72] Phillips A. (2015). Unforbidden Pleasures.

[73] Clark A. (2019). Beyond desire? Agency, choice, and the predictive mind. Australas. J. Philos..

[74] Yon D., Heyes C., Press C. (2020). Beliefs and desires in the predictive brain. Nat. Commun..

[75] Lee K.M., Ferreira-Santos F., Satpute A.B. (2021). Predictive processing models and affective neuroscience. Neurosci. Biobehav. Rev..

[76] Kruglanski A.W., Jasko K., Friston K. (2020). All Thinking is ‘Wishful’ Thinking. Trends Cogn. Sci..

[77] Ondobaka S., Kilner J., Friston K. (2017). The role of interoceptive inference in theory of mind. Brain Cogn..

[78] Friston K., Frith C. (2015). A Duet for one. Conscious. Cogn..

[79] Solms M. (2021). Hidden Spring: A Journey to the Source of Consciousness.

